# Networks of worry—towards a connectivity-based signature of late-life worry using higher criticism

**DOI:** 10.1038/s41398-021-01648-5

**Published:** 2021-10-28

**Authors:** Andrew R. Gerlach, Helmet T. Karim, Joseph Kazan, Howard J. Aizenstein, Robert T. Krafty, Carmen Andreescu

**Affiliations:** 1 Department of Psychiatry, University of Pittsburgh, Pittsburgh, PA USA; 2 Department of Bioengineering, University of Pittsburgh, Pittsburgh, PA USA; 3 Department of Biostatistics and Bioinformatics, Emory University, Atlanta, GA USA

**Keywords:** Psychiatric disorders, Neuroscience

## Abstract

Severe worry is a complex transdiagnostic phenotype independently associated with increased morbidity, including cognitive impairment and cardiovascular diseases. We investigated the neurobiological basis of worry in older adults by analyzing resting state fMRI using a large-scale network-based approach. We collected resting fMRI on 77 participants (>50 years old) with varying worry severity. We computed region-wise connectivity across the default mode network (DMN), anterior salience network, and left executive control network. All 22,366 correlations were regressed on worry severity and adjusted for age, sex, race, education, disease burden, depression, anxiety, rumination, and neuroticism. We employed higher criticism, a second-level method of significance testing for rare and weak features, to reveal the functional connectivity patterns associated with worry. The analysis suggests that worry has a complex, yet distinct signature associated with resting state functional connectivity. Intra-connectivities and inter-connectivities of the DMN comprise the dominant contribution. The anterior cingulate, temporal lobe, and thalamus are heavily represented with overwhelmingly negative association with worry. The prefrontal regions are also strongly represented with a mix of positive and negative associations with worry. Identifying the most salient connections may be useful for targeted interventions for reducing morbidity associated with severe worry in older adults.

## Introduction

Severe worry, defined as “a chain of thoughts and images, negatively affect-laden and relatively uncontrollable” [1], is one of the core components in the symptomatology of multiple anxiety and depressive disorders [2]. Epidemiological studies show that anxiety disorders are the most common neuropsychiatric disorders with a lifetime prevalence of up to 28.8% [3], and about one in four adults living with generalized anxiety report first onset after the age of 50 [4]. Although worry is traditionally associated with generalized anxiety disorder (GAD), recent studies showed that only 20% of older adults with severe worry qualify for a GAD diagnosis [5]. Studies have shown an independent association between worry severity and the development of hypertension and coronary heart disease [6]. Worry was also specifically associated with cognitive decline. A longitudinal study indicated that at two-year follow-up, older adults with severe worry had significant memory decline compared with those with low worry [7]. In a recent study combining neuroimaging data with machine learning, our group has reported that severe worry was significantly associated with accelerated brain aging [8]. Additionally, worry was associated with several symptoms of psychological distress, independently of the primary psychiatric diagnosis [9]. Considering the transdiagnostic nature of worry, coupled with its high prevalence and broad consequences for both physical and mental health, it is important to understand the neurobiological basis of worry in order to develop effective treatment approaches.

Over the past decade, the focus of brain analyses has shifted from localization to a network-based perspective. This is particularly important for complex constructs such as worry, whose neurobiological correlates are likely to be more intricate and rely on several brain networks and network interactions [10]. In particular, the triple network interaction model suggests that affective dysregulation may be linked to intra-network and inter-network connectivity involving the default mode network (DMN), anterior salience network (ASN), and central executive network (sometimes also called the executive control network, or ECN) [11]. Furthermore, this model has been extended to transdiagnostic phenotypes reflecting the heterogeneity of anxiety and depression, suggesting that the inconsistent neuroimaging results associated with traditional categorical diagnoses would be difficult to translate to individual cases and, consequently, to help guiding treatment choices [12]. These three canonical brain networks and their interactions have been implicated in the neural basis of several anxiety disorders and phenotypes [13, 14], though very few studies have specifically focused on worry, and even less so on late-life worry. The DMN and ASN in particular have been a major focus as the neurobiological correlates of anxiety and worry, though the results have not always been consistent [15, 16].

Aging is associated with a reconfiguration of both structural and functional connectivity [17], and multiple studies have reported on age-related changes in the canonical brain networks [17–20]. There is however a limited amount of data regarding the association of late-life worry with functional connectivity indices [21, 22]. In late-life, greater worry severity has been shown to be correlated with lower resting state functional connectivity between the posterior cingulate cortex (PCC) and medial prefrontal cortex (mPFC) [22] and greater connectivity between right anterior insula and precuneus [23]. In younger adults, worry has been associated with greater resting state connectivity between the right amygdala and right superior frontal gyrus, right anterior cingulate cortex (ACC), and right supramarginal gyrus after worry induction [24]. Connectivity with the PCC, ACC, and precuneus has been negatively correlated with anxious apprehension, one of the affective facets of severe worry [25]. A recent study on repetitive negative thinking, which includes both worry and rumination, found greater resting state functional connectivity between amygdala and PFC regions/precuneus associated with greater worry severity [26]. A focused review identified the dorsal ACC (dACC) and dorsomedial PFC (dmPFC) as key regions for conscious threat appraisal, one of the cognitive facets of worry [27]. A recent meta-analysis of GAD versus non-GAD controls found a preponderance of results centered on the dorsolateral PFC (dlPFC), ACC, amygdala, and hippocampus [28]. Of these four major regions, only the amygdala showed consistent results with greater functional connectivity for participants with GAD [13, 29]. The insula, PCC, precuneus, precentral gyrus, superior temporal gyrus, ventrolateral PFC, orbitofrontal cortex (OFC), and cerebellum are mentioned as other common regions of interest, again with many mixed results. A recent meta-analysis of task-based fMRI studies investigating worry notes a similar inconsistency across the literature [30]. Across these studies of the neurobiological basis of anxiety and worry, three common themes are 1) a lack of consistent or reproducible results, 2) a lack of studies investigating late-life worry, and 3) limited explorations of connectivity that most often has been related to a specific seed/network, thus offering an incomplete model of the neural basis of worry.

To the best of our knowledge, the current study represents the largest investigation of the neurobiological basis of worry in older adults using resting state fMRI. Given our previous publication on the association between worry and accelerated brain aging [8], and in the context of the increased risk severe worry poses to cardiovascular and cerebrovascular disease [6, 31, 32], it is imperative to characterize the whole brain connectivity signature of late-life worry. This is particularly significant considering the transdiagnostic nature of worry and its prevalence across various late-life psychopathologies [33]. Developing a better understanding of the neuroimaging characteristics implicated in the pathology of worry is essential for the development of interventions targeting worry-specific regions and networks. In our study, we use the statistical approach of higher criticism, which, to the best of our knowledge, has not been used for neuroimaging analysis. We believe this approach is optimally suited to identify the multiple, but sometimes subtle, changes in brain connectivity that may underlie the worry process. Higher criticism, described in more detail in the methods section, is a second-level method of significance testing for rare and weak features capable of detecting the presence of a brain-wide signature of worry and identifying the connectivities most likely to contribute to such a neural signature. Using resting state fMRI, we investigated the association between worry severity and node-wise functional connectivity of the DMN, ASN, and left ECN (LECN) and employed higher criticism to discriminate the most relevant connections.

## Materials and methods

### Participants and study design

This data was collected as part of the Functional Neuroanatomy Correlates of Worry in Older Adults study (R01 MH108509) at the University of Pittsburgh. The study proposed to enroll 150 subjects in order to have 80% power to detect a correlation between worry and four pre-specified seed-ROI functional connectivity indices with an individual *R*^2^ of 7% at the 5% significance level while using Bonferroni correction to control for multiple comparisons. This midterm analysis seeks to answer a different question and to analyze associations between worry and a more comprehensive collection of connectivities with a potentially weak and sparse signal. A basic power analysis using the HDDesign package [34] for R based on higher criticism [35] indicates that an effect size of 0.1 with 100 important features is detectable with 81 participants, decreasing rapidly as the number of important features increases. We recruited 110 participants over the age of 50 into a cross-sectional study through Pitt+Me (a research portal at the University of Pittsburgh), in-person recommendations, flyers, and radio/television advertisements. Participants with and without DSM-V diagnoses of anxiety and mood disorders were recruited to maintain balance across the spectrum of worry. Diagnoses were assessed using the structured clinical interview for DSM-V (SCID). Of the 110 participants, 28 (25%) had a DSM-V diagnosis of GAD, 27 (25%) were diagnosed with any anxiety order other than GAD over their lifetime, and 25 (23%) were diagnosed with MDD over their lifetime. We excluded participants with any form of psychosis or bipolar disorder, mild cognitive impairment or dementia, a history of substance abuse within the last 6 months, and participants who use medications with known effects on the fMRI signal (e.g., theophylline, aminophylline). Participants were psychotropic-free, having undergone a 2-week washout if previously on an antidepressant (6 weeks for fluoxetine). This study was approved by the University of Pittsburgh Institutional Review Board. All participants gave written informed consent prior to participating in the study.

### Assessments

Basic demographic information was collected for each participant: age, sex, race, and education. Several psychological measures were also collected: depression was assessed with the Montgomery-Åsberg Depression Rating Scale (MADRS) [36]; overall anxiety was assessed with the Hamilton Anxiety Rating Scale (HARS) [37]; worry was self-assessed with the Penn State Worry Questionnaire (PSWQ)[38]; rumination was self-assessed with the Rumination Style Questionnaire (RSQ) [39]; neuroticism was self-assessed with the subscale from the Five Factor Inventory (NEO-FFI) [40]; and perceived stress was self-assessed with the Perceived Stress Scale (PSS) [41]. Additionally, disease burden was evaluated with the Cumulative Illness Rating Scale for Geriatrics (CIRS-G) [42].

### MRI data acquisition

Imaging data was collected at the Magnetic Resonance Research Center at the University of Pittsburgh with a 3T Siemens Prisma scanner using a 32-channel head coil. Resting state data was acquired over a single 6-min interval while participants were directed to fixate on a white crosshair in the center of a black screen and told to not fall asleep. Whole-brain T2*-weighted BOLD images were acquired axially using gradient echo-planar imaging (EPI) sequence with the following parameters: repetition time (TR)/echo time (TE) = 1000/30 ms, flip angle (FA) 45°, FOV 96 × 96 with 60 axial slices, 2.3 mm^3^ isotropic resolution with 2.3 mm slice gap, and multiband acceleration factor of 5. Three anatomical images were also collected. Whole-brain T1-weighted images were acquired sagittally using a magnetization prepared rapid gradient echo (MPRAGE) sequence with TR/TE = 2400/2.22 ms, FA 8°, FOV 300 × 320 with 208 sagittal slices, 0.8 mm^3^ isotropic resolution with no slice gap (total time 6.63 min). A sagittal, whole-brain T2-weighted sampling perfection with application optimized contrasts using different flip angle evolution (SPACE) image was collected with TR/TE = 3200 ms/563 ms, FA 120°, FOV = 320 × 300 with 208 slices, 0.8 mm^3^ isotropic resolution with no slice gap, and generalized autocalibrating partial parallel acquisition (GRAPPA) with acceleration factor of 2 (total time 5.95 min). An axial, whole-brain T2-weighted fluid-attenuated inversion recovery (FLAIR) was collected with TR/TE = 10,000/91 ms, FA = 135°, inversion time (TI) = 2500 ms, FOV = 320 × 320 with 104 slices, 0.8 mm × 0.8 mm × 1.6 mm resolution with no slice gap, and GRAPPA with acceleration factor of 2 (total time 5.95 min). Participants were in the MR scanner for approximately 45–60 min as we collected other MRI data as well (not presented).

### MR image preprocessing

The structural MRI data was processed with the Statistical Parametric Mapping (SPM12) toolbox [43] in MatLab 2018b (MathWorks, Natick, MA). All interpolation was performed with a 4th degree B-spline and the similarity metric used for coregistration between different image types was normalized mutual information. The T2 SPACE and FLAIR images were first coregistered to the MPRAGE image. All three were input into the segmentation routine to generate the deformation field to standard MNI space and bias-corrected images, as well as probability maps for six tissue classes [44]. Because of the high burden of white matter hyperintensities, we adjusted the number of Gaussians used to identify white matter to two, which improves identification of gray and white matter [45]. The MPRAGE image was then skull-stripped so it could be used to coregister the functional images—this was done by generating an intracranial volume mask (thresholding gray matter, white matter, and cerebrospinal by 0.1 then conducting image filling and image closing in MatLab). This process also generates a deformation field, which is used to normalize fMRI to MNI space.

The resting state fMRI was processed with SPM12 in MatLab and the FMRIB Software Library v6.0 Brain Extraction Tool (FSL BET) [46]. Images were slice time corrected and motion-corrected to the mean functional image with a rigid body transformation. Motion parameters in each of the three translational and rotational directions were estimated during motion correction. The functional images were skull-stripped using BET, visually inspected, and coregistered to the MPRAGE image before being transformed to MNI space using the deformation field generated in structural processing. The images were spatially smoothed with an 8 mm Gaussian kernel and motion-induced spikes were removed using the BrainWavelet toolbox [47]. Finally, the following were regressed out of each voxel: six rigid-body motion parameters, first five principal components of the white matter and cerebral spinal fluid, and sinusoids corresponding to unwanted frequencies outside of the resting state band 0.008 and 0.15 Hz of interest for resting state analysis (i.e., a band-pass filter). By doing this in one step, we do not reintroduce artifact/noise into our signal [48]. We used the ArtRepair toolbox [49] to calculate several subject-level parameters of motion including max translations, average root mean squared, average scan-to-scan motion, and percent of headjerks (>0.5 mm movements).

### Resting state functional connectivity

The processed images were used to calculate the functional connectivity with in-house scripts in MatLab. Network templates for the DMN, ASN, and LECN were generated by selecting prominent hubs of the networks, determining their central coordinate from the AAL3 brain atlas definition of the nodes [50], obtaining the resting state connectivity maps for those coordinates from the Neurosynth.org database of 1000 participants [51], and combining the relevant brain maps.

The hubs utilized for network definition were the bilateral mPFC and PCC for the DMN, the bilateral dACC and insula for the ASN, and the left dlPFC and inferior parietal lobe for the LECN. Coordinates in MNI space for these nodes are shown in Supplementary Table 1 and displayed in Supplementary Fig. 1. A threshold of 0.1 (positive correlations only) was used to binarize the brain maps. The resulting network maps, also shown in Supplementary Fig. 1, were then segmented with the AAL3 definitions to generate network-specific ROIs. The cerebellum labels were removed from consideration due to insufficient coverage in the resting state data, leaving 79 nodes in the DMN, 78 nodes in the ASN, and 55 nodes in the LECN. Note that many regions are present in more than one network, though the network-specific maps may differ if they do not cover the full extent of that region. The mean time signal was calculated for each ROI and the connectivity between the pair-wise regions was quantified with the Pearson correlation, resulting in a 212 by 212 correlation matrix. We refer to these 22,366 correlations as connectivities.

### Statistical analysis

Statistical analysis was performed in R v4.0.2 [52] via in-house code (available upon request). A Fisher Z-Transform was applied to the connectivities to transform the correlation distribution to a normal distribution. A linear model was fit to each correlation with PSWQ as the predictor of interest and age, sex, race, education, CIRS-G, MADRS, HARS, PSS, RSQ, and NEO-FFI as confounds. The MADRS score was converted to a categorical variable with a threshold of 14 since the distribution of MADRS scores is highly skewed. The relevant result was a regression coefficient for worry with associated *z*-value and *p*-value for each of the 22,366 connectivities. In trying to assess significant connectivities associated with worry, we encounter a challenging multiple comparisons problem. As we expected few of the connectivities to be significantly associated with worry, and we did not anticipate those significant associations to be particularly strong, we required a statistical test capable of detection in such an environment. For this, we leveraged higher criticism (HC) [53, 54] as a second-level significance test specifically formulated for the rare/weak regime.

While popular in the genomics field [55], this is the first application of HC to neuroimaging data as far as we are aware. In light of this, we believe it is worthwhile to provide some context for HC, particularly with respect to how its results may be interpreted. The fundamental question HC addresses is: “given some large number of statistical tests, is there enough group significance to believe that the results did not occur by random chance?” In our application of HC, the statistical tests are the 22,366 regressions. Given no association with worry, we would expect the regression coefficients to follow a normal distribution centered about 0, of which an average of 1118 would be significant at the 95% confidence level. HC tests whether our data is consistent with this global null hypothesis by looking at the distribution as a whole. Rather than considering the regression coefficients directly, the resultant *p*-values are compared to the null (uniform) distribution, which allows optimal incorporation of both frequency and strength information. The *p*-values are arranged in ascending order, *p*_i_ for *i*∈(1,…,22,366), and at each point *i* the empirical *p*-values are compared to the expected null (uniform) distribution:

$${\rm{HC}}_{\rm{i}} = \sqrt n \frac{{i/n-p_{\rm{i}}}}{{\sqrt {i/n(1 - i/n)} }},$$

This statistic is similar to a *z*-score with *p*_i_ the observed variable, *i*/*n* the expected value, and $\frac{i}{n}(1 - \frac{i}{n})/n$ the variance of a binomial distribution with probability of success *i*/*n*. For large *n*, the central limit theorem dictates that this statistic approaches a standard normal distribution, and hence a value of greater than 2 would indicate a statistically significant deviation from the null distribution of *p*-values at the 95% confidence level. Or in our application, it would provide significant evidence to reject the global null hypothesis that resting state functional connectivity has no association with worry.

As a secondary outcome, HC identifies the group of tests which provide the most evidence against the group null hypothesis. This is accomplished by using the maximum of the HC statistic to define a cutoff point for selection of the tests contributing the most evidence against the null hypothesis. It is important to note that HC does not offer any predefined type I error control at the individual level. This stands in contrast to typical analyses that control for the number or rate of type I errors at a given confidence level with family wise error or false discovery rate procedures, respectively. This may also be considered somewhat analogous to ANOVA, which can identify if there is a statistically significant difference between group means but not which mean(s) differ(s) significantly, though HC does identify the most likely contributors to the observed effect. To gain further insight on the relative importance of each connectivity, 1000 bootstrap iterations were performed tracking which (if any) connectivities were identified by HC as most likely contributors to the observed effect. For each iteration, the subjects were sampled with replacement, linear models were fit for all 22,366 connectivities, and HC was applied to the results. If the maximum HC statistic was less than 2, none of the connectivities were tallied. Otherwise, the connectivities contributing to the maximum HC statistic were tallied. Note that this is not a test of statistical significance (since weak effects would not be expected to be identified in 95% of the iterations), and merely a means to rank the importance of the connectivities.

To summarize, HC can be used to identify the presence of global functional connectivity significantly associated with worry (i.e., does worry have a neurobiological signature observable through resting state functional connectivity analysis?). If there is indeed a global difference, HC also provides the individual connectivities that most contribute to this determination, though it does not offer strict control of false positives in this step.

## Results

Out of the 110 participants, 77 were included in the analysis; their characteristics are summarized in Table 1. Seven participants failed preprocessing steps, one had significant signal dropout in the resting state scan, and three participants had excessive head motion (defined as >20% of volumes with head jerks). Out of the remaining participants, 22 were missing demographic data necessary to perform the linear regressions, leaving 77 participants with complete resting state data as well as clinical and demographic data. The following measures were missing: education (4), CIRS-G (7), PSWQ (1), MADRS (6), HARS (3), RSQ (8), NEO-FFI (12). The demographic data is compared between the participants included in the analysis and those excluded for missing data (data presented in Supplementary Table 2). Significant differences exist for race, education, PSWQ, HARS, RSQ, and NEO-FFI at the 95% confidence level, driven primarily by the 11 subjects excluded for scanner-related reasons.

**Table 1:** Summary of participant demographics (*n* = 77).

Measure	Mean	Std. Dev.
Age (years)	61.8	8.2
Sex (no. female)	48 (62%)	–
Race (W/B/MR)	87 (88%), 8 (10%), 1 (1%)	–
Education	16.0	2.3
CIRS-G	3.7	3.5
PSWQ	48.2	14.6
MADRS	7.4	7.9
HARS	7.4	5.7
RSQ	37.0	12.9
NEO-FFI	19.2	9.9

Linear models were fit to predict all 22,366 connectivities from worry, as well as the demographic and psychiatric confounds. The empirical *p*-values associated with the regression coefficients for worry were input to HC. The maximum HC statistic was 5.16, providing very strong evidence against the null hypothesis that resting state functional connectivity is not associated with worry severity as measured by the PSWQ. Thus, there is a distinct resting state signature of worry independent of other demographic and clinical factors.

There were 154 connectivities identified by HC as contributing the most evidence for a neural signature of worry. Included are both positive associations with worry (i.e., greater worry severity associated with greater connectivity between two nodes) and negative associations with worry between and across the DMN, ASN, and LECN. Table 2 provides a summary of the distribution of these connectivities. The absolute beta coefficients and *p*-values for the relevant connectivities are shown in Supplementary Table 3, ordered by their prevalence in 1000 bootstrap iterations.

**Table 2:** Distribution of relevant connectivities identified by HC within and between the networks.

Network	Positive association with worry	Negative association with worry
DMN	11	22
ASN	3	12
LECN	8	8
DMN-ASN	12	22
ASN-LECN	14	5
LECN-DMN	24	13

Greater worry is associated with greater connectivity (positive associations) in the LECN-DMN network connectivities while greater worry is associated with lower connectivity in the intra-DMN and DMN-ASN connectivities.

**Fig. 1:**
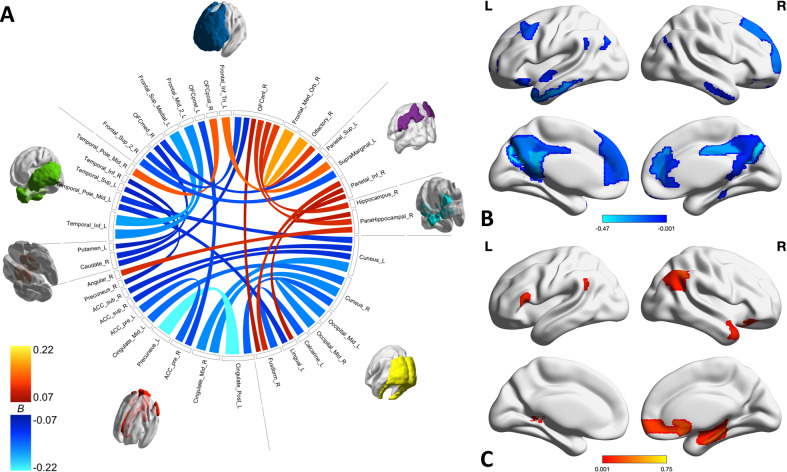
Within DMN connectivities and regions associated with worry. **A** Chord diagram shows individual connections identified by HC with both weight and color indicating the strength of the association with worry severity (positive values/warm colors indicate stronger connectivity between regions is associated with greater worry, negative values/cool colors indicate stronger connectivity between region sis associated with less worry). **B**, **C** Brain maps show the sum of the negative (**B**) and positive (**C**) associations with worry for each region. Negative associations with worry are heavily concentrated in the bilateral ACC, PCC, and cuneus as well as the left temporal lobe. Prefrontal regions show a mix of positive and negative associations with worry.

**Fig. 2:**
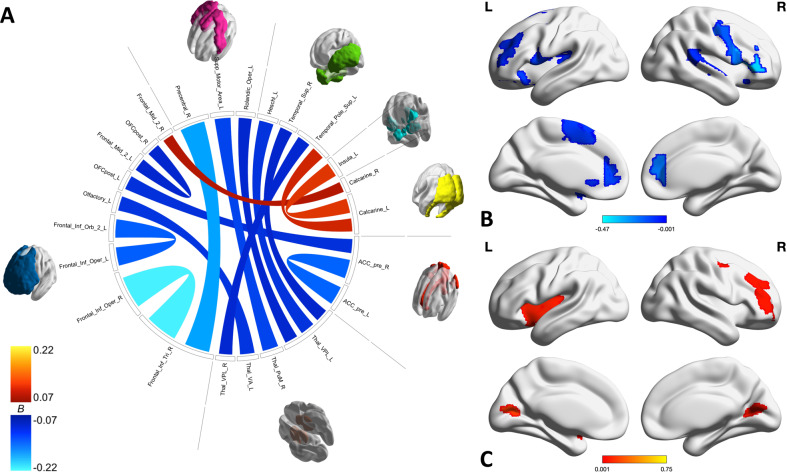
Within ASN connectivities and regions associated with worry. **A** Chord diagram shows individual connections identified by HC with both weight and color indicating the strength of the association with worry severity. **B**, **C** Brain maps show the sum of the negative (**B**) and positive (**C**) associations with worry for each region. Negative associations with worry dominate, especially in the ACC, thalamus, OFC, and other inferior frontal regions.

**Fig. 3:**
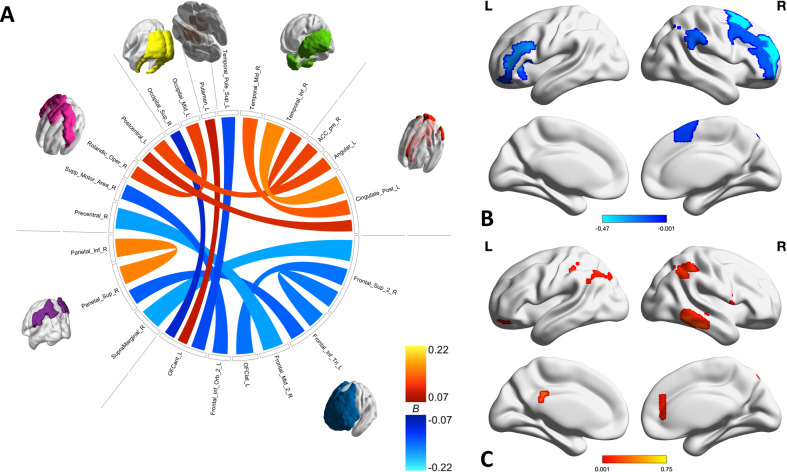
Within LECN connectivities and regions associated with worry. **A** Chord diagram shows individual connections identified by HC with both weight and color indicating the strength of the association with worry severity. **B**, **C** Brain maps show the sum of the negative (**B**) and positive (**C**) associations with worry for each region. Negative associations with worry all involve the PFC while positive associations are focused in the left PCC and right temporal lobe.

Furthermore, we explore the group of relevant connectivities within and between each network. Toward this goal, we employ two graphical summaries of the connectivities in Figs. 1–3 and Supplementary Figs. 2–4: chord diagrams to show the individual connections and brain maps to show a weighted degree centrality. For the chord diagrams, produced with the R package *circlize* [56], the individual ROIs are sorted into default mode, limbic, parietal, prefrontal, sensorimotor, subcortical, temporal, and visual groups. Both the color and weight of the line connecting any two ROIs indicate the magnitude of the regression coefficient, or how strongly that connectivity is associated with worry. Warm colors are used for positive associations with worry (i.e., greater connectivity and greater worry) and cool colors for negative associations (i.e., lower connectivity and greater worry). The brain maps, visualized with BrainNet Viewer [57], are specific to positive or negative associations to avoid cancellation. The intensity of a region is simply the sum of the regression coefficients standardized with respect to worry for positive or negative connectivities involving that region. In other words, a region can achieve a high intensity through its number of relevant connectivities or through the strength of its connectivities’ association with worry.

Lastly, we verified that the HC approach was appropriate for this analysis by checking for a network-wide association with worry severity using template based rotation [58]. Using standard multiple comparisons correction (statistical non-parametric mapping toolbox [http://www.nisox.org/Software/SnPM13/] with cluster-forming threshold of *p* = .001 with 10,000 permutations and controlling the cluster-wise family-wise error rate *p* < .05), there were no significant associations with worry in the DMN, ASN, or LECN, providing further justification for the HC approach to identify rare/weak features of interest.

The DMN contains the most relevant connections of the networks, as shown in Fig. 1, two thirds of which are negatively associated with worry (greater worry, lower connectivity). Lower connectivities associated with greater worry are heavily concentrated in the cingulate (both ACC and PCC), cuneus, and temporal lobes. Greater connectivities associated with greater worry are concentrated in the right hippocampus and parahippocampus, the parietal lobe, and the frontal regions, particularly the OFC. The frontal regions show a particularly robust and complex pattern of connectivity, including both positive and negative positive associations with worry.

The ASN contains the fewest relevant connectivities of the networks, as shown in Fig. 2. The relevant connectivities are dominated by negative associations with worry severity, particularly in the OFC, inferior frontal regions, ACC, and thalamus. Of the three connectivities with a positive association with worry, all involve the calcarine sulcus.

The LECN contains a more balanced proportion of connectivities with positive and negative associations with worry, as shown in Fig. 3. The positively associated connectivities are focused in the left PCC and right temporal lobe while the negatively associated connectivities are focused in the frontal cortex.

The DMN-ASN interconnectivity, shown in Supplementary Fig. 2, contains many relevant connections, with a preference toward negative associations with worry. Notably, the cingulate and cuneus are heavily represented with almost uniformly negative associations with worry. Meanwhile, connectivities with the parietal lobe are strongly skewed toward positive associations with worry. The frontal regions again show a mix of positive and negative associations with worry, with substantial representation by the OFC.

The ASN-LECN network interconnectivity, shown in Supplementary Fig. 3, contains the most positive associations with worry, particularly in the frontal regions and temporal lobes, but also in the cingulate.

The LECN-DMN network interconnectivity, shown in Supplementary Fig. 4, presents several relevant connections. With the exception of the temporal lobes, which are heavily represented and almost uniformly carry a positive association with worry, the mix of positive and negative associations defies a simple summary. However, we note that in addition to the temporal lobe, frontal, visual, and default mode regions are prominently represented.

## Discussion

To summarize, our study identified a complex pattern of connectivity within and between the canonical brain networks associated with worry severity in older adults. The relevant DMN connectivities had a predominately negative correlation with worry severity spread throughout multiple regions, with a few relevant positive correlations involving the right OFC, hippocampus/parahippocampus and the inferior parietal gyrus. The ASN has the fewest relevant in-network connectivities, and these were overwhelmingly negative (higher worry associated with lower connectivity with regions such the OFC, ACC, thalamus, and inferior frontal gyrus). The LECN showed a positive association with predominantly posterior cortical areas and a negative association with predominantly frontal areas. The between-network connectivity displays a few notable associations: DMN–ASN interconnectivity is mostly negatively associated with worry severity (more worry, less connectivity), particularly in the anterior and mid-cingulate, while DMN-LECN and ASN-LECN interconnectivity is mostly positively associated with worry severity, especially in the prefrontal and temporal regions, though there’s a relative paucity of results in the ASN–LECN.

Overall, the complex clinical phenomenology of worry (blend of negative-affective and perseverative-cognitive processes) is reflected at the neural level with engagement of both paralimbic/other subcortical and multiple integrative cortical regions, consistent with the conclusions of meta-analyses on GAD (of which worry is a primary component) [28] and perseverative cognition (i.e., worry and rumination) [59]. This intricate neurobiological signature combined with the observational bias induced by methodological choices, particularly seed choice, may explain the inconsistencies in the literature noted in the introduction. Thus, there are several studies in the literature describing the association of DMN hyperconnectivity with perseverative negative cognitive processes [60, 61], others describing hypoconnectivity in the DMN [22, 25], and some that find no association at all within the DMN [26]. While the heterogeneity in results may be connected to other methodological differences (e.g., use of different scales like PSWQ versus State-Trait Anxiety Scale), we may speculate that most of the results offer an accurate but ultimately incomplete presentation of the neurobiological basis of worry.

The intricate nature of worry’s neural signature does not preclude the extraction of salient features, however. We noted a bounty of connectivities involving the ACC negatively associated with worry, which has a well-established role in conflict monitoring [62] and cognitive reappraisal [63]. This suggests that a more connected ACC may be better “wired” to effectively implement emotion regulation strategies and downplay severe worry. Concurrently, the perseverative cognitive aspects inherent in the worry process appear more prominent as worry become more severe, as evidenced by the predominantly positive association of worry severity with LECN within and between connectivity. This aspect is highly relevant for future potential interventions such as transcranial magnetic stimulation, as they may target regions associated with the difficult-to-control feature of severe worry and, consequently, downgrade the worry process to an easier to regulate negative affect.

Beyond the results of this study, the higher criticism methodology may be of unique interest to the field of neuroimaging. The use of HC allowed us to rigorously test for the presence of a neurobiological signature, a step that is often taken for granted, and unveil distinct patterns of connectivity that otherwise would have been obfuscated by traditional methodologies. It is able to accomplish this for rare and weak effects by offering a fundamentally different approach to the multiple comparisons problem. While our application fits satisfyingly in the HC framework, there may be other creative uses for it in the field of neuroimaging, particularly as an exploratory or feature reduction tool.

Our study has several limitations. While the moderate sample size provided sufficient power to determine that there is a neurobiological correlate of worry observable in resting state fMRI, a larger sample size would allow us to more precisely pinpoint the primary contributor to that signature. The focus on worry later in life (>50) also limits the generalizability of the results to a wider age range. Our group has previously demonstrated an age-by-anxiety interaction on resting state functional connectivity [22], so we expect that the neural signature of worry would also demonstrate an age dependence. Other limitations are the inclusion of anxiety and rumination (and to a lesser extent depression and neuroticism) as confounds in the regression model, which may obscure aspects of worry intimately tied to those measures, and the exclusive focus on intrinsic connectivity. Furthermore, the resting state scan was acquired over a single 6-min interval. A study exploring the effect of scan length found that 12-min scans improve intra-session and inter-session reliability of resting state connectivity [64], while 6-min scans represent the lower end of the acceptable spectrum, which may limit the robustness of our results. However, the intraclass correlation coefficient for network connections was 0.6 for 6-min scans, representing moderate reliability. The effect of brain aging on the intricate model presented above will be the subject of a separate report. We excluded 33 participants from the total sample, who differed significantly on race, education, PSWQ, HARS, RSQ, and NEO-FFI. While the exclusion criteria were nominally objective, this represents a substantial and unfortunate source of bias. Finally, the use of HC may be regarded as both a limitation and a strength. While it allows us to establish the presence of a neurobiological basis of worry, it limits our ability to localize that signature in a manner that offers strict type I error control. However, the benefits of this approach outweigh the drawbacks, especially for the exploration of complex phenomena such as worry.

In conclusion, we use a novel approach to describe the within and inter-network connectivity associations of worry in late-life, a fine-grain approach that allows us to shed light on the neural complexity underlying severe worry.

## Supplementary information

Supplement

Supplementary Table 3

Supplementary Figure 1

Supplementary Figure 2

Supplementary Figure 3

Supplementary Figure 4

## Data Availability

De-identified data is available to researchers upon request.
