# Supplementary material for: Networks of worry—towards a connectivity-based signature of late-life worry using higher criticism
Source: Transl Psychiatry. 2021 Oct 28;11:550. doi: 10.1038/s41398-021-01648-5 (PMC8553743; doi:10.1038/s41398-021-01648-5)
Supplement: Supplementary file 1 — Supplement [file 41398_2021_1648_MOESM1_ESM.docx]

**Supplementary Table 1. MNI location of network hubs. Parenthetical superscripts are for cross-reference with Supplementary Figure 1**

| **Network** | **Node** | **Central MNI Coordinate**  **(x, y, z)** |
| --- | --- | --- |
| DMN | left mPFC ^(1)^ | (-5, 59, -8) |
|  | right mPFC ^(2)^ | (8, 49, -8) |
|  | left PCC ^(3)^ | (-3, -46, 25) |
|  | right PCC ^(4)^ | (8, -44, 24) |
| ASN | left dACC ^(5)^ | (-4, 32, 22) |
|  | right dACC ^(6)^ | (9, 30, 20) |
|  | left Insula ^(7)^ | (-39, 13, -3) |
|  | right Insula ^(8)^ | (43, 13, -5) |
| LECN | left dlPFC ^(9)^ | (-30, 48, 22) |
|  | left IPL ^(10)^ | (-46, -46, 44) |

**Supplementary Table 2**. Comparison of measures between analyzed participants (n=77) and those excluded for unusable scans or missing data (n=33)

| **Measure** | **Mean (Analyzed)** | **Mean (Excluded)** | ***p* Value** |
| --- | --- | --- | --- |
| Age (yrs) | 61.8 | 59.2 | 0.133 |
| Sex (no. female) | 48 (62%) | 22 (67%) | 0.669 |
| Race (W/B/HPI/MR) | 87 (88%), 8 (10%), 0 (0%), 1 (1%) | 24 (86%), 8 (24%), 1 (3%), 0 (0%) | 0.043† |
| Education | 16.0 | 14.4 | 0.003 |
| CIRSG | 3.7 | 5.2 | 0.052 |
| PSWQ | 48.2 | 54.5 | 0.029 |
| MADRS | 7.4 | 8.2 | 0.134 |
| HARS | 7.4 | 11.8 | 0.005 |
| RSQ | 37.0 | 42.8 | 0.047 |
| NEO-FFI | 19.2 | 24.4 | 0.040 |

† Black (B), Hawaiian Pacific Islander (HPI), and multiracial (MR) categories were combined

**Supplementary Table 3**. List of connectivities identified by HC as providing the most evidence for a resting state basis of worry, the coefficient from the regression of worry on the connectivity standardized with respect to worry (*B*), and the associated p-value ordered by their bootstrap prevalence. Note that the three networks exhibit significant overlap, so many regions appear in more than one network. The regions rarely cover the entire anatomical node, so the network-specific regions typically differ and thus the connection between two regions may appear in the table more than once, though always with different network designations.

(Table attached in spreadsheet)

**Supplementary Figure 1**. Network maps for the DMN (red), ASN (green), and LECN (blue). Nodes used to seed the networks are indicated with black dots; numbers correspond to the parenthetical superscripts of the nodes in Supplementary Table 1. Network overlaps between the DMN and ASN (yellow), ASN and LECN (cyan), and LECN and DMN (magenta) are also shown.

**Supplementary Figure 2**. Between DMN and ASN connectivities and regions associated with worry. (A) Chord diagram shows individual connections identified by HC with both weight and color indicating the strength of the association with worry severity. (B-C) Brain maps show the sum of the negative (B) and positive (C) associations with worry for each region. Negative associations with worry are heavily concentrated throughout the cingulate cortex and cuneus while connectivities involving the parietal lobe are predominately positive. The frontal regions, particularly the OFC, are heavily represented with a mix of positive and negative associations with worry.

**Supplementary Figure 3**. Between ASN and LECN connectivities and regions associated with worry. (A) Chord diagram shows individual connections identified by HC with both weight and color indicating the strength of the association with worry severity. (B-C) Brain maps show the sum of the negative (B) and positive (C) associations with worry for each region. Positive associations with worry abound, particularly in the frontal regions and temporal lobes.

**Supplementary Figure 4**. Between LECN and DMN connectivities and regions associated with worry. (A) Chord diagram shows individual connections identified by HC with both weight and color indicating the strength of the association with worry severity. (B-C) Brain maps show the sum of the negative (B) and positive (C) associations with worry for each region. Positive associations with worry are abundant in the temporal lobes, while other regions show a more elusive pattern of positive and negative associations.
