# Supplementary material for: Networks of worry—towards a connectivity-based signature of late-life worry using higher criticism
Source: Transl Psychiatry. 2021 Oct 28;11:550. doi: 10.1038/s41398-021-01648-5 (PMC8553743; doi:10.1038/s41398-021-01648-5)
Supplement: Supplementary file 2 — Supplementary Table 3 [file 41398_2021_1648_MOESM2_ESM.pdf]

Network1	Region1	Network2	Region2	Regression Coefficient	*P* -value	Bootstrap Prevalence
LECN	Temporal_Inf_R	LECN	Cingulate_Post_L	0.01049	0.00001	56.7%
LECN	Cingulate_Post_L	DMN	Cuneus_L	-0.01215	0.00011	39.1%
LECN	Cingulate_Post_L	DMN	Cuneus_R	-0.00990	0.00029	35.3%
LECN	Temporal_Mid_R	LECN	Cingulate_Post_L	0.00832	0.00017	35.3%
LECN	Frontal_Mid_2_R	DMN	Fusiform_R	0.00602	0.00017	34.2%
DMN	Cuneus_R	ASN	Cingulate_Mid_L	-0.01002	0.00028	33.5%
DMN	Cuneus_R	ASN	Occipital_Sup_R	0.01507	0.00032	31.8%
LECN	Frontal_Inf_Orb_2_R	DMN	Fusiform_R	0.00548	0.00017	31.8%
LECN	Temporal_Inf_R	DMN	Temporal_Mid_L	0.00782	0.00062	31.8%
DMN	Parietal_Inf_R	DMN	Fusiform_R	0.00605	0.00041	30.8%
LECN	Temporal_Inf_R	ASN	Cingulate_Post_L	0.00820	0.00029	30.4%
DMN	Cuneus_R	DMN	Cingulate_Mid_L	-0.00920	0.00112	30.2%
LECN	Temporal_Inf_R	DMN	Cingulate_Post_L	0.00977	0.00029	30.1%
DMN	N_Acc_R	ASN	Calcarine_L	-0.00554	0.00063	29.2%
DMN	Cuneus_R	ASN	Cingulate_Post_L	-0.01006	0.00083	29.1%
LECN	Temporal_Inf_R	LECN	ACC_pre_R	0.00730	0.00052	28.3%
DMN	ParaHippocampal_R	ASN	Thal_VA_L	0.00792	0.00026	28.0%
LECN	Temporal_Inf_R	DMN	Temporal_Mid_R	0.01065	0.00055	27.8%
LECN	Parietal_Inf_R	DMN	Fusiform_R	0.00536	0.00083	27.7%
DMN	Temporal_Pole_Sup_R	ASN	Rolandic_Oper_L	-0.00904	0.00044	27.3%
DMN	ParaHippocampal_R	DMN	OFCant_R	0.00648	0.00032	27.1%
LECN	Temporal_Mid_R	DMN	Temporal_Mid_R	0.01297	0.00048	26.7%
DMN	Cuneus_R	DMN	Cingulate_Mid_R	-0.01059	0.00120	26.6%
LECN	Frontal_Inf_Tri_L	DMN	Frontal_Sup_2_R	-0.01125	0.00045	26.4%
LECN	Temporal_Pole_Sup_L	ASN	Frontal_Inf_Oper_L	-0.01211	0.00231	25.2%
LECN	Cingulate_Mid_L	DMN	Cuneus_R	-0.00931	0.00180	25.2%
DMN	Fusiform_R	ASN	Frontal_Mid_2_R	0.00563	0.00076	24.9%
ASN	Thal_PuM_R	ASN	Supp_Motor_Area_L	-0.00746	0.00120	24.9%
DMN	ParaHippocampal_L	ASN	Cingulate_Mid_R	0.00740	0.00089	24.7%
LECN	OFClat_L	DMN	Frontal_Sup_2_R	-0.01010	0.00035	24.6%
LECN	SupraMarginal_R	DMN	Temporal_Sup_L	0.00617	0.00252	24.3%

ASN	Thal_VPL_L	ASN	Heschl_L	-0.00674	0.00120	23.6%
LECN	Frontal_Sup_2_R	DMN	Calcarine_L	0.00878	0.00090	23.3%
DMN	Temporal_Pole_Mid_R	DMN	OFCpost_R	0.00826	0.00090	23.3%
ASN	Thal_VA_L	ASN	Olfactory_L	-0.00679	0.00128	23.2%
LECN	Frontal_Sup_2_R	DMN	Calcarine_R	0.00816	0.00112	23.1%
DMN	Temporal_Inf_R	DMN	Putamen_L	-0.00538	0.00162	22.7%
DMN	Olfactory_R	DMN	Frontal_Med_Orb_R	0.01164	0.00088	22.6%
DMN	Cuneus_R	ASN	Cingulate_Mid_R	-0.00997	0.00191	22.3%
DMN	Temporal_Sup_L	ASN	SupraMarginal_L	0.00610	0.00111	22.3%
DMN	Cuneus_R	DMN	Cingulate_Post_L	-0.00873	0.00169	22.2%
LECN	Postcentral_L	DMN	Fusiform_R	-0.00523	0.00226	22.0%
LECN	Temporal_Inf_R	ASN	ACC_pre_R	0.00761	0.00144	21.5%
LECN	Temporal_Inf_R	DMN	ACC_pre_R	0.00761	0.00144	21.5%
ASN	Frontal_Inf_Orb_2_L	ASN	Frontal_Inf_Oper_L	-0.00860	0.00160	21.4%
DMN	OFCant_R	DMN	Hippocampus_R	0.00628	0.00093	21.3%
DMN	Parietal_Sup_L	DMN	OFCmed_R	-0.00702	0.00101	21.3%
LECN	Occipital_Sup_R	LECN	OFCant_L	-0.00591	0.00144	21.3%
DMN	OFCant_R	DMN	Caudate_R	-0.00629	0.00131	21.0%
DMN	Cuneus_L	DMN	ACC_sup_R	-0.00667	0.00180	20.5%
DMN	Temporal_Pole_Mid_L	DMN	Cuneus_L	-0.00699	0.00167	20.3%
ASN	OFCpost_L	ASN	ACC_pre_R	-0.00658	0.00207	20.0%
DMN	ACC_pre_R	ASN	OFCpost_L	-0.00658	0.00207	20.0%
LECN	OFCant_R	DMN	Fusiform_R	0.00511	0.00154	19.9%
LECN	Postcentral_L	LECN	Cingulate_Post_L	0.00636	0.00264	19.9%
LECN	OFCant_L	ASN	Putamen_L	0.00555	0.00176	19.6%
DMN	Cingulate_Mid_R	ASN	Precuneus_L	-0.01069	0.00127	19.2%
ASN	Thal_VPL_L	ASN	Temporal_Sup_R	-0.00598	0.00165	19.1%
LECN	Parietal_Sup_R	LECN	Frontal_Inf_Tri_L	-0.00865	0.00141	19.1%
LECN	Temporal_Mid_R	ASN	Cingulate_Post_L	0.00713	0.00128	18.8%
LECN	Temporal_Mid_L	ASN	Temporal_Sup_L	0.00856	0.00146	18.8%
LECN	Frontal_Inf_Oper_L	ASN	Frontal_Inf_Oper_R	0.01323	0.00315	18.7%
ASN	Thal_VPL_R	ASN	Temporal_Pole_Sup_L	-0.00618	0.00167	18.7%
LECN	Cingulate_Mid_R	ASN	Insula_L	0.01250	0.00228	18.6%

LECN	Rolandic_Oper_R	LECN	Occipital_Mid_L	0.00729	0.00122	18.5%
LECN	Frontal_Inf_Orb_2_L	ASN	Frontal_Inf_Oper_L	-0.00899	0.00247	18.4%
DMN	Parietal_Inf_R	DMN	ParaHippocampal_R	0.00531	0.00363	18.3%
DMN	Temporal_Inf_L	ASN	Calcarine_L	0.00588	0.00198	18.1%
LECN	SupraMarginal_L	DMN	Frontal_Sup_2_R	-0.01013	0.00083	18.1%
DMN	N_Acc_R	ASN	Calcarine_R	-0.00446	0.00400	17.9%
ASN	Thal_VPL_L	ASN	Rolandic_Oper_L	-0.00612	0.00159	17.8%
DMN	Olfactory_R	DMN	OFCant_R	0.00712	0.00306	17.8%
LECN	Rolandic_Oper_L	DMN	OFCpost_R	-0.00614	0.00191	17.8%
LECN	Temporal_Mid_R	DMN	ParaHippocampal_R	0.00567	0.00379	17.8%
ASN	Temporal_Pole_Sup_L	ASN	Calcarine_L	0.00605	0.00246	17.7%
LECN	OFClat_L	ASN	Rolandic_Oper_R	0.00652	0.00160	17.6%
LECN	Angular_R	DMN	ACC_sup_L	0.00614	0.00323	17.6%
LECN	Temporal_Inf_R	DMN	Angular_L	0.00833	0.00245	17.6%
ASN	Frontal_Mid_2_R	ASN	Calcarine_R	0.00493	0.00231	17.5%
ASN	Frontal_Inf_Tri_R	ASN	Frontal_Inf_Oper_R	-0.01523	0.00218	17.4%
DMN	SupraMarginal_L	DMN	Frontal_Sup_2_R	-0.00814	0.00272	17.4%
LECN	SupraMarginal_R	LECN	Frontal_Sup_2_R	-0.01186	0.00218	17.4%
DMN	OFCpost_L	ASN	ACC_pre_R	-0.00746	0.00283	17.3%
DMN	Olfactory_L	ASN	Parietal_Inf_R	0.00647	0.00223	17.3%
DMN	OFCpost_L	DMN	ACC_pre_R	-0.00746	0.00283	17.3%
LECN	Precentral_R	DMN	Occipital_Sup_L	0.00682	0.00219	17.3%
DMN	Temporal_Sup_L	DMN	Precuneus_R	-0.00562	0.00188	17.3%
DMN	Temporal_Mid_R	ASN	Parietal_Inf_L	0.00883	0.00178	17.2%
DMN	Cuneus_L	ASN	ACC_sub_R	-0.00681	0.00165	17.1%
DMN	Temporal_Sup_L	ASN	Olfactory_R	0.00731	0.00394	17.1%
LECN	Cingulate_Post_L	DMN	Frontal_Mid_2_R	0.00795	0.00300	17.1%
DMN	Temporal_Inf_L	DMN	Frontal_Mid_2_L	-0.01072	0.00387	17.0%
DMN	Fusiform_R	ASN	Frontal_Inf_Oper_L	-0.00485	0.00284	16.9%
DMN	ParaHippocampal_R	DMN	Angular_R	0.00639	0.00363	16.9%
DMN	Occipital_Mid_R	DMN	Occipital_Mid_L	-0.01035	0.00158	16.7%
LECN	Putamen_L	LECN	OFCant_L	0.00548	0.00293	16.7%
ASN	ACC_pre_R	ASN	ACC_pre_L	-0.00883	0.00361	16.6%

DMN	ACC_pre_R	ASN	ACC_pre_L	-0.00883	0.00361	16.6%
DMN	SupraMarginal_L	DMN	Frontal_Inf_Tri_L	0.00907	0.00132	16.6%
DMN	SupraMarginal_L	ASN	Frontal_Sup_2_R	-0.00903	0.00283	16.4%
DMN	OFCpost_R	ASN	Rolandic_Oper_L	-0.00772	0.00236	16.4%
LECN	Pallidum_L	ASN	Thal_VPL_R	-0.00540	0.00180	16.4%
DMN	OFCant_R	ASN	Putamen_R	0.00494	0.00214	16.3%
DMN	ACC_pre_L	ASN	ACC_pre_R	-0.00879	0.00383	16.2%
DMN	OFCant_R	ASN	Temporal_Sup_R	0.00575	0.00292	16.2%
DMN	ACC_pre_R	DMN	ACC_pre_L	-0.00879	0.00383	16.2%
DMN	Temporal_Pole_Mid_L	DMN	Lingual_L	-0.00665	0.00297	16.0%
LECN	Frontal_Inf_Oper_L	ASN	Frontal_Inf_Tri_L	0.01191	0.00283	15.9%
LECN	Occipital_Sup_R	DMN	Frontal_Sup_Medial_L	0.00819	0.00323	15.8%
DMN	OFCant_R	DMN	Fusiform_R	0.00517	0.00277	15.7%
LECN	Frontal_Mid_2_L	DMN	OFCmed_R	-0.00632	0.00279	15.7%
LECN	Frontal_Sup_2_R	DMN	Fusiform_R	0.00554	0.00276	15.6%
ASN	Precentral_R	ASN	Frontal_Inf_Tri_R	-0.01171	0.00216	15.4%
DMN	OFCmed_R	ASN	Frontal_Sup_2_L	-0.00926	0.00179	15.2%
DMN	Temporal_Sup_L	ASN	Caudate_L	-0.00623	0.00202	15.1%
LECN	Frontal_Sup_2_R	ASN	Cuneus_L	0.00638	0.00339	15.0%
DMN	Fusiform_R	DMN	Calcarine_L	-0.00855	0.00157	15.0%
DMN	Thal_PuM_R	ASN	Supp_Motor_Area_L	-0.00648	0.00351	14.9%
DMN	Temporal_Inf_L	DMN	Frontal_Sup_Medial_L	-0.01164	0.00254	14.9%
LECN	OFClat_L	DMN	Temporal_Sup_L	0.00473	0.00253	14.9%
DMN	Cuneus_L	DMN	Cingulate_Mid_R	-0.01076	0.00181	14.8%
DMN	Parietal_Inf_R	DMN	Lingual_L	0.00517	0.00339	14.8%
LECN	OFCant_L	ASN	OFCpost_R	-0.00604	0.00202	14.6%
LECN	Frontal_Sup_2_L	DMN	OFCmed_R	-0.00842	0.00250	14.6%
LECN	Temporal_Pole_Sup_L	LECN	OFCant_L	-0.00764	0.00258	14.6%
DMN	Cuneus_L	ASN	Cingulate_Mid_R	-0.01080	0.00292	14.5%
LECN	Frontal_Mid_2_L	DMN	Thal_PuM_L	0.00564	0.00311	14.5%
DMN	OFCmed_R	DMN	Caudate_R	-0.00638	0.00404	14.1%
LECN	Putamen_L	ASN	Insula_R	-0.00627	0.00184	14.0%
LECN	Postcentral_L	LECN	Angular_L	0.00759	0.00360	13.8%

LECN	OFClat_L	ASN	Paracentral_Lobule_R	0.00594	0.00220	13.7%
DMN	Precuneus_L	DMN	Cingulate_Post_L	-0.01573	0.00201	13.6%
LECN	Insula_R	DMN	Occipital_Mid_R	0.00698	0.00242	13.6%
DMN	Thal_PuM_R	ASN	Temporal_Sup_L	-0.00645	0.00387	13.3%
LECN	Temporal_Inf_R	DMN	Rectus_R	0.00701	0.00295	13.3%
LECN	Frontal_Inf_Tri_R	ASN	Temporal_Mid_R	0.00953	0.00214	13.2%
LECN	OFClat_L	LECN	Frontal_Sup_2_R	-0.00955	0.00206	13.0%
LECN	Parietal_Sup_R	LECN	Parietal_Inf_R	0.01024	0.00352	13.0%
DMN	OFCant_R	DMN	Frontal_Sup_2_R	-0.00746	0.00400	12.9%
DMN	Cuneus_L	DMN	ACC_sub_R	-0.00651	0.00308	12.8%
LECN	Frontal_Sup_2_R	LECN	Frontal_Inf_Tri_L	-0.00976	0.00353	12.8%
LECN	Cingulate_Post_L	DMN	ACC_sup_R	-0.00511	0.00390	12.7%
LECN	Precentral_R	LECN	Frontal_Mid_2_R	-0.01155	0.00277	12.5%
LECN	Temporal_Mid_R	DMN	Cingulate_Post_L	0.00881	0.00366	12.2%
ASN	OFCpost_R	ASN	Frontal_Mid_2_L	-0.00602	0.00374	12.1%
DMN	Cingulate_Mid_L	ASN	Precuneus_L	-0.00986	0.00359	12.1%
LECN	Caudate_L	DMN	Temporal_Sup_L	-0.00572	0.00383	11.3%
LECN	OFClat_L	ASN	Temporal_Sup_R	0.00608	0.00292	11.0%
DMN	Cuneus_L	ASN	ACC_sub_L	-0.00644	0.00344	10.9%
LECN	Supp_Motor_Area_R	LECN	Frontal_Inf_Orb_2_L	-0.00845	0.00341	10.9%
LECN	OFClat_L	DMN	Supp_Motor_Area_L	-0.00725	0.00396	10.8%
ASN	Insula_L	ASN	Calcarine_L	0.00680	0.00370	10.6%
LECN	OFCant_R	ASN	Putamen_R	0.00534	0.00335	10.3%
DMN	Frontal_Sup_Medial_L	ASN	Parietal_Sup_L	0.00799	0.00390	9.3%
