# Supplementary figures and images for: Networks of worry—towards a connectivity-based signature of late-life worry using higher criticism
Source: Transl Psychiatry. 2021 Oct 28;11:550. doi: 10.1038/s41398-021-01648-5 (PMC8553743; doi:10.1038/s41398-021-01648-5)

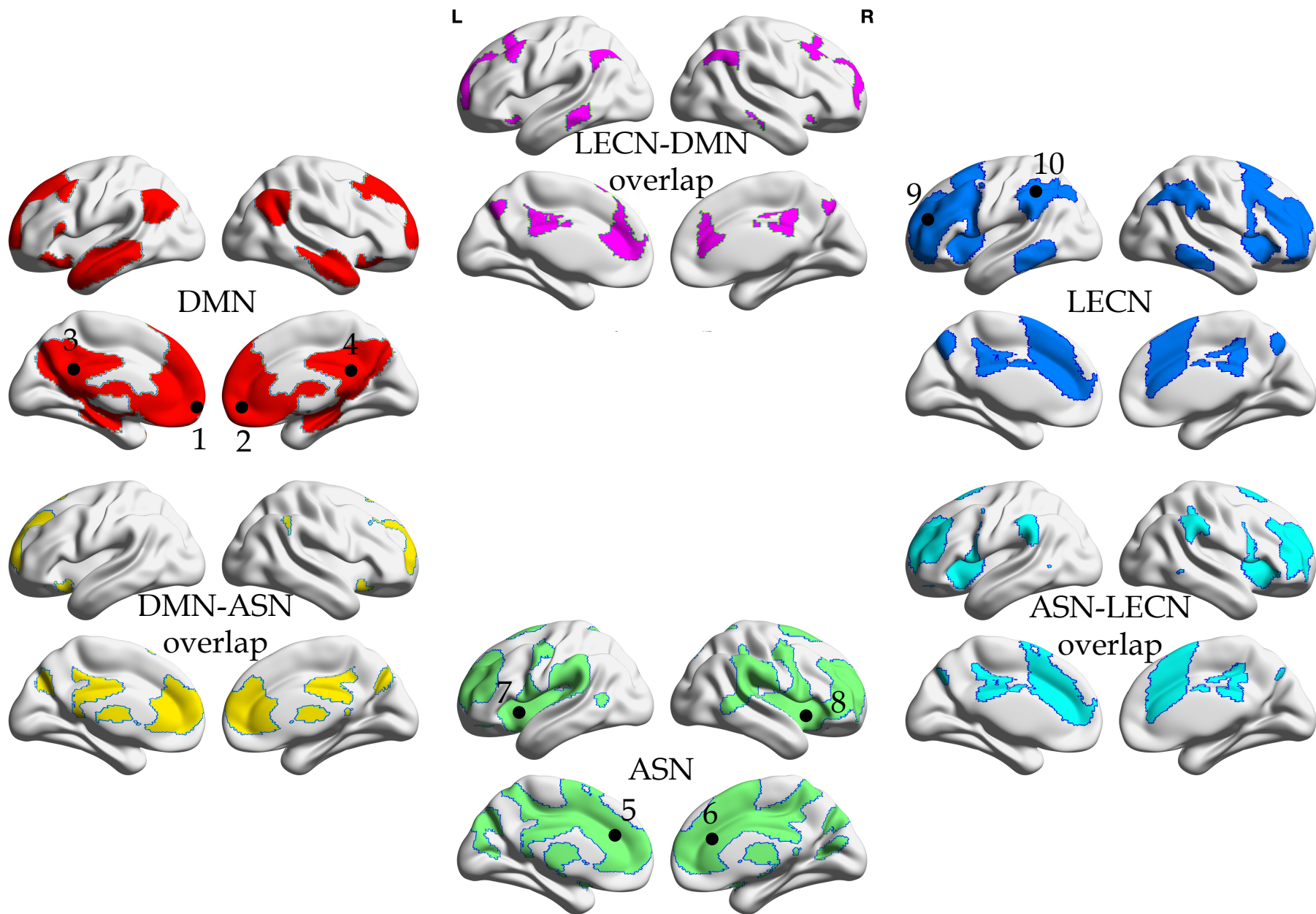

Supplement: Supplementary file 3 — Supplementary Figure 1 [file 41398_2021_1648_MOESM3_ESM.pdf]

**A**

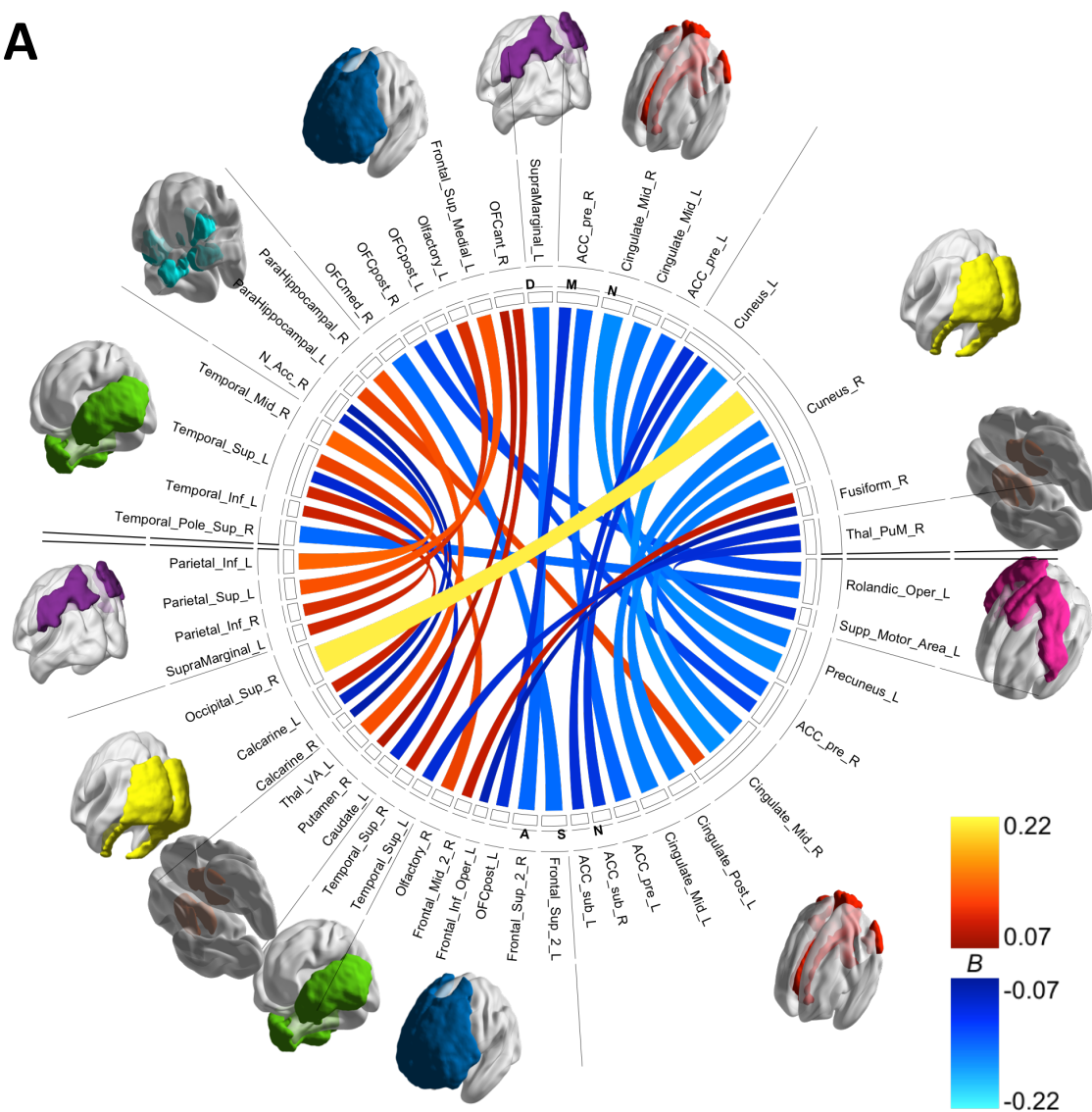

**L**

**R**

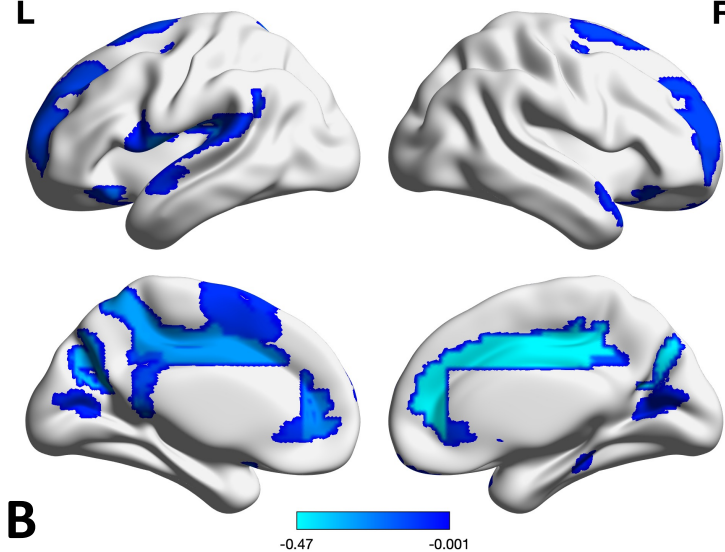

**B**

**L**

**R**

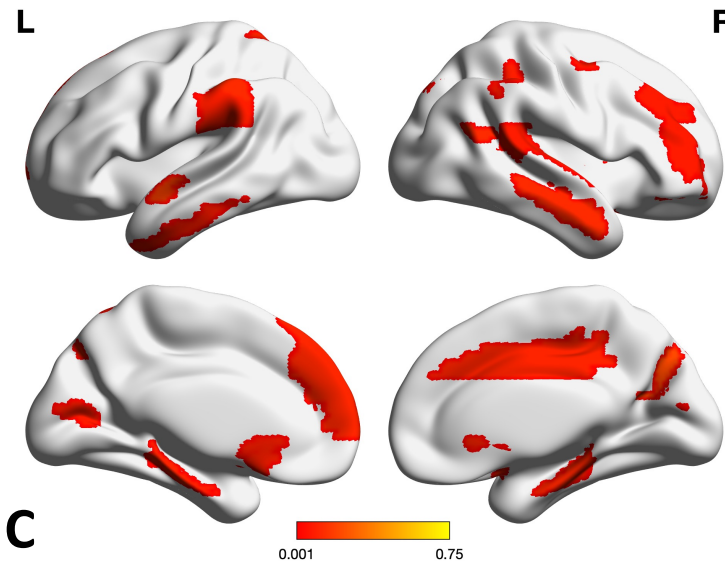

**C**

Supplement: Supplementary file 4 — Supplementary Figure 2 [file 41398_2021_1648_MOESM4_ESM.pdf]

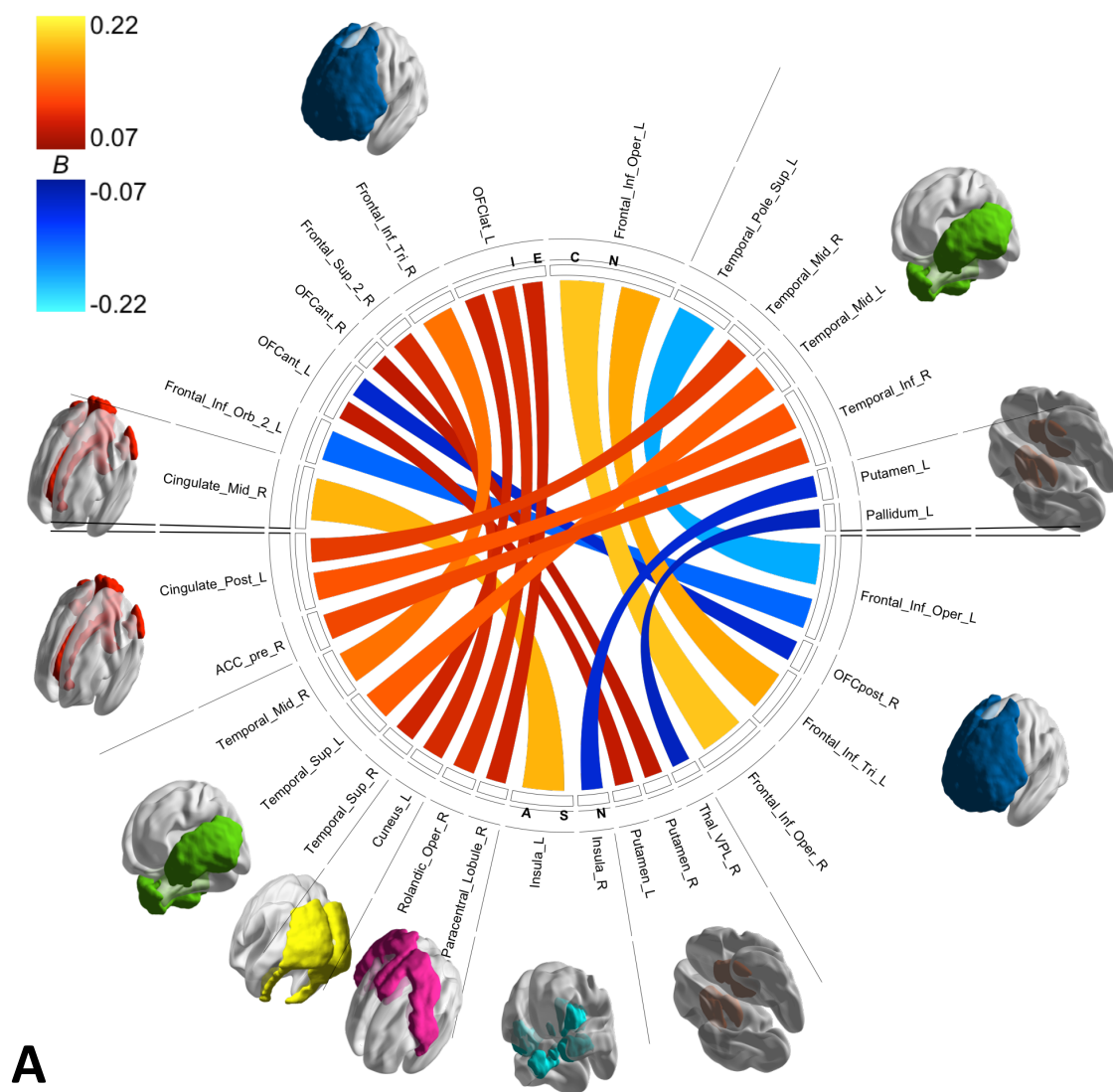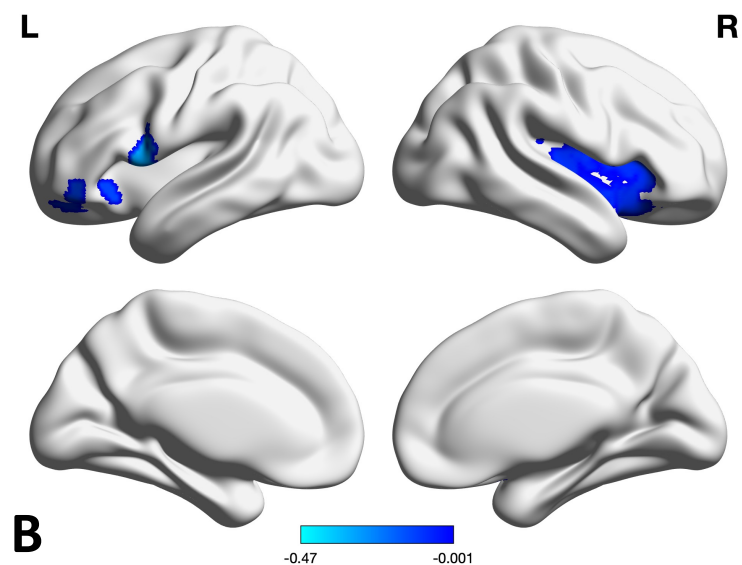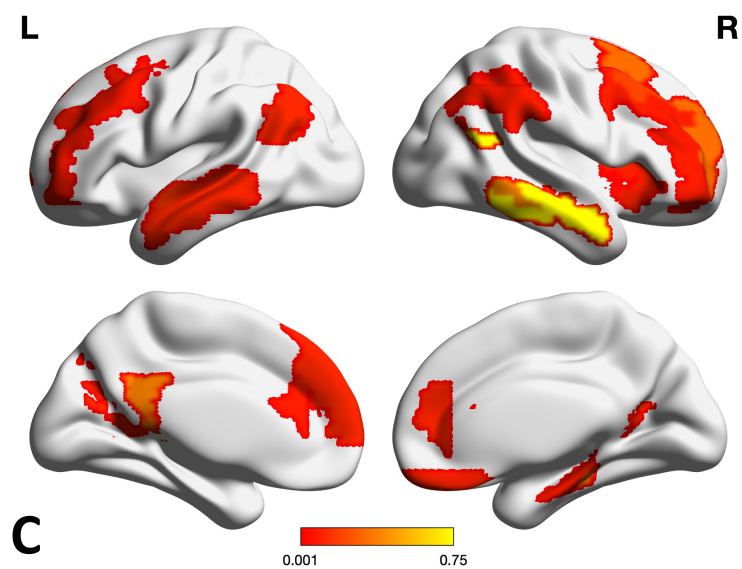

Supplement: Supplementary file 5 — Supplementary Figure 3 [file 41398_2021_1648_MOESM5_ESM.pdf]

**A**

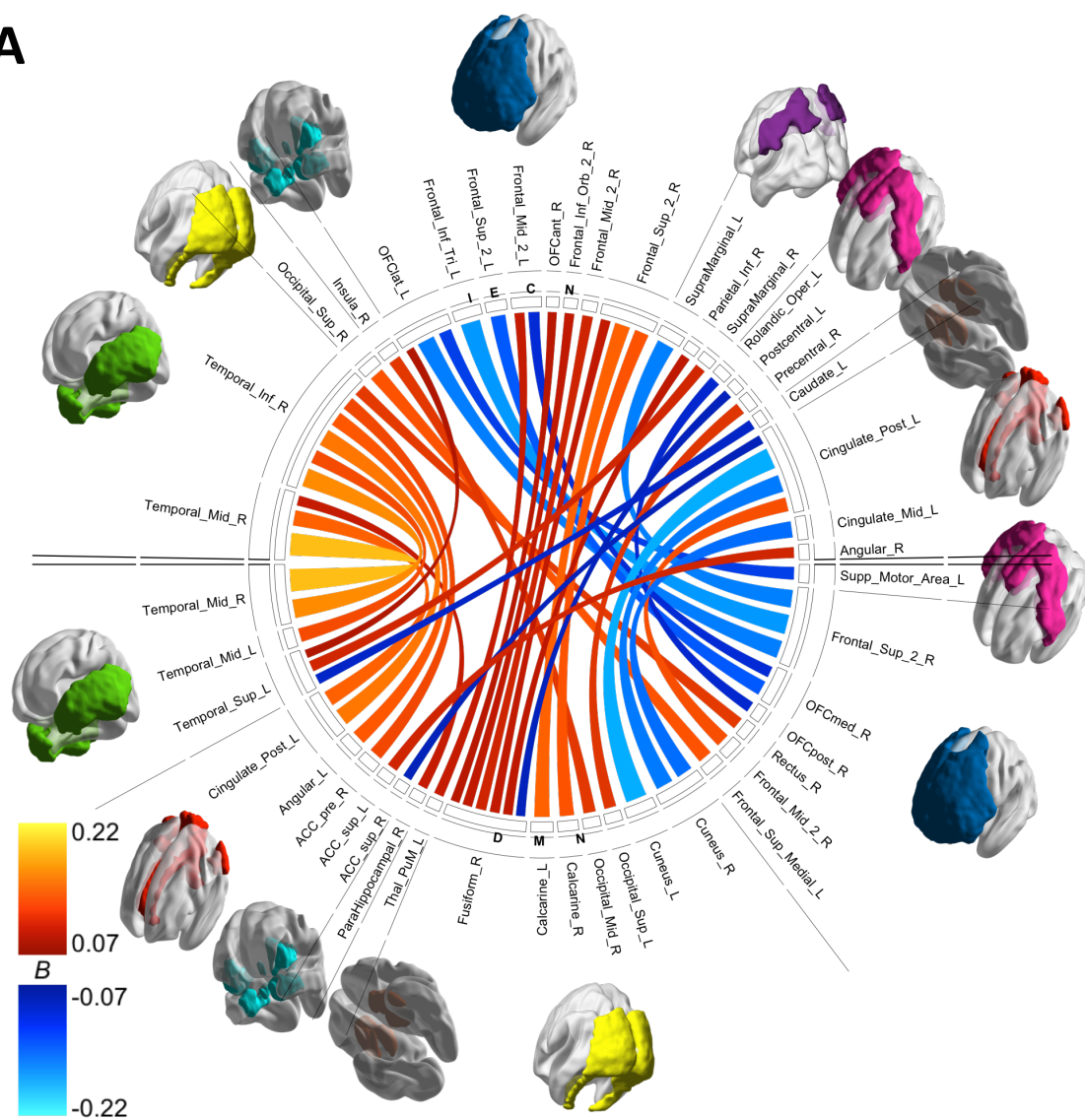

**L**

**R**

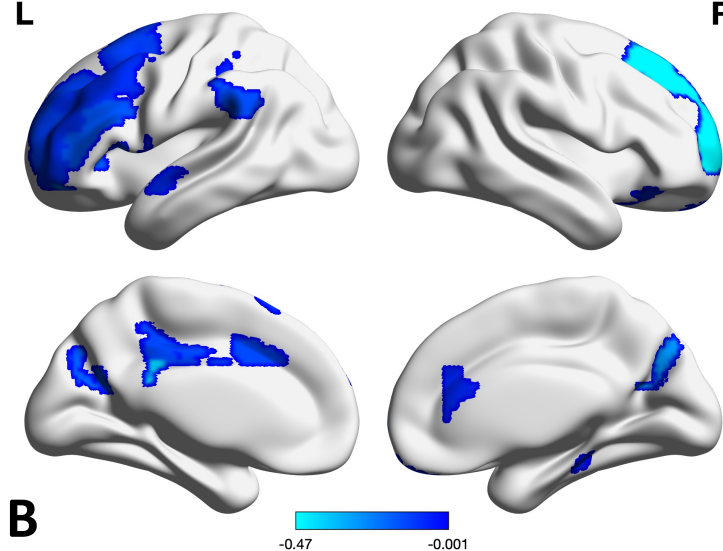

**B**

**L**

**R**

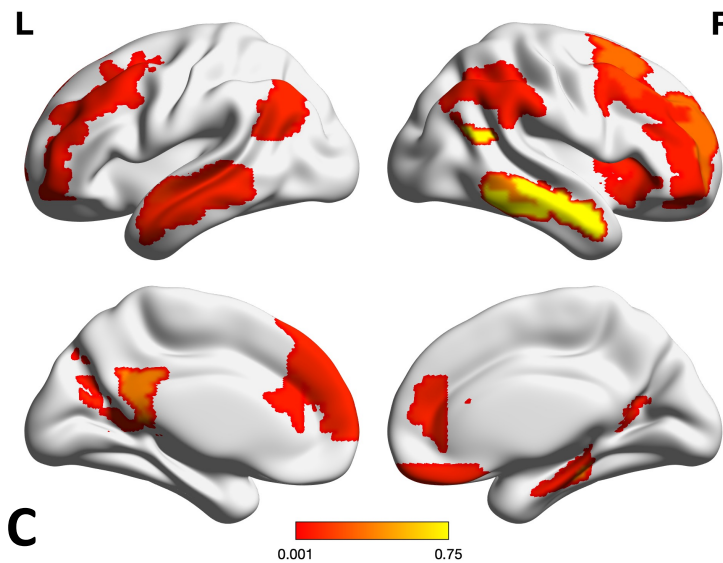

**C**

Supplement: Supplementary file 6 — Supplementary Figure 4 [file 41398_2021_1648_MOESM6_ESM.pdf]
